# Supplementary material for: The use of technology for social interaction by people with dementia: A scoping review
Source: PLOS Digit Health. 2022 Jun 6;1(6):e0000053. doi: 10.1371/journal.pdig.0000053 (PMC9931370; doi:10.1371/journal.pdig.0000053)
Supplement: S1 Table — (DOCX) [file pdig.0000053.s002.docx]

S2 Table. EMBASE Search Strategy

|  | Database | Search term |
| --- | --- | --- |
| 37 | EMBASE | (dementia).ti,ab |
| 38 | EMBASE | exp DEMENTIA/ |
| 39 | EMBASE | exp "CREUTZFELDT JAKOB DISEASE"/ |
| 40 | EMBASE | (Alzheimer OR Alzheimers OR alzheimer's).ti,ab |
| 41 | EMBASE | (Creutzfeldt-Jakob).ti,ab |
| 42 | EMBASE | ("Huntington's disease" OR "Huntington chorea" OR "Huntington's disease").ti,ab |
| 43 | EMBASE | (37 OR 38 OR 39 OR 40 OR 41 OR 42) |
| 44 | EMBASE | (immersive).ti,ab |
| 45 | EMBASE | "VIRTUAL REALITY"/ |
| 46 | EMBASE | "COMPUTER INTERFACE"/ |
| 47 | EMBASE | "COMPUTER ASSISTED THERAPY"/ |
| 48 | EMBASE | (virtual).ti,ab |
| 49 | EMBASE | ("augmented reality").ti,ab |
| 50 | EMBASE | (technology OR technologies OR technological).ti,ab |
| 51 | EMBASE | (robot*).ti,ab |
| 52 | EMBASE | (multimedia).ti,ab |
| 53 | EMBASE | ("photo book").ti,ab |
| 54 | EMBASE | (tv OR television).ti,ab |
| 55 | EMBASE | (media).ti,ab |
| 56 | EMBASE | (computer*).ti,ab |
| 57 | EMBASE | (digital).ti,ab |
| 58 | EMBASE | ("personalised music").ti,ab |
| 59 | EMBASE | TECHNOLOGY/ |
| 60 | EMBASE | ROBOTICS/ |
| 61 | EMBASE | (ebook OR "electronic book").ti,ab |
| 62 | EMBASE | (phone OR phones OR telephone OR telephones).ti,ab |
| 63 | EMBASE | (ipad* OR iphone*).ti,ab |
| 64 | EMBASE | (facetime OR whatsapp OR messenger).ti,ab |
| 65 | EMBASE | ("mobile app" OR "mobile apps").ti,ab |
| 67 | EMBASE | exp "MOBILE PHONE"/ OR TELEPHONE/ OR "TEXT MESSAGING"/ OR "SOCIAL MEDIA"/ |
| 68 | EMBASE | (smartphone*).ti,ab |
| 69 | EMBASE | (44 OR 45 OR 46 OR 47 OR 48 OR 49 OR 50 OR 51 OR 52 OR 53 OR 54 OR 55 OR 56 OR 57 OR 58 OR 59 OR 60 OR 61 OR 62 OR 63 OR 64 OR 65 OR 67 OR 68) |
| 70 | EMBASE | (43 AND 69) |
| 71 | EMBASE | (Connectedness OR connection).ti,ab |
| 72 | EMBASE | (Communication).ti,ab |
| 73 | EMBASE | (interaction).ti,ab |
| 74 | EMBASE | (conversation OR conversations).ti,ab |
| 75 | EMBASE | (friendship OR friendships).ti,ab |
| 76 | EMBASE | (language).ti,ab |
| 77 | EMBASE | (reminiscence).ti,ab |
| 78 | EMBASE | (loneliness OR lonely).ti,ab |
| 79 | EMBASE | (social OR socially).ti,ab |
| 80 | EMBASE | (network*).ti,ab |
| 81 | EMBASE | (relationships).ti,ab |
| 82 | EMBASE | LONELINESS/ OR exp "SOCIAL ISOLATION"/ |
| 83 | EMBASE | (isolated OR isolation).ti,ab |
| 84 | EMBASE | FRIENDSHIP/ OR exp "SOCIAL NETWORK"/ |
| 85 | EMBASE | (71 OR 72 OR 73 OR 74 OR 75 OR 76 OR 77 OR 78 OR 79 OR 80 OR 81 OR 82 OR 83 OR 84) |
| 86 | EMBASE | (70 AND 85) |
| 88 | EMBASE | (internet*).ti,ab |
| 89 | EMBASE | ("i‐pad" OR "i‐phone" OR ipod OR "i‐pod").ti,ab |
| 90 | EMBASE | (Facebook).ti,ab |
| 91 | EMBASE | ("text messag*" OR texting).ti,ab |
| 92 | EMBASE | (YouTube OR podcast).ti,ab |
| 93 | EMBASE | (web*).ti,ab |
| 94 | EMBASE | (88 OR 89 OR 90 OR 91 OR 92 OR 93) |
| 95 | EMBASE | (43 AND 85 AND 94) |
| 96 | EMBASE | (69 OR 94) |
| 97 | EMBASE | (43 AND 85 AND 96) |
