# Supplementary material for: The use of technology for social interaction by people with dementia: A scoping review
Source: PLOS Digit Health. 2022 Jun 6;1(6):e0000053. doi: 10.1371/journal.pdig.0000053 (PMC9931370; doi:10.1371/journal.pdig.0000053)
Supplement: Attachment — Submitted filename: Response to reviewers.docx [file pdig.0000053.s003.docx]

Dr Merryn Anderson

Wonford House

Dryden Road

Exeter

EX2 5AF

Dear Shlomo Berkovsky, Section Editor, PLOS Digital Health

Re. PDIG-D-22-00008: The use of technology for social interaction by people with dementia: a scoping review

Thank you for your email and comments regarding the manuscript I submitted. Below are my responses to the editor and reviewers comments.

Please let me know if there are any more changes needed

Kind regards

Dr Merryn Anderson

Academic Clinical Fellow, University of Exeter

**Response to reviewers**

*Editor’s comments*

Please also consider expanding the search to technical repositories, such as IEEE Xplore and ACM Digital Library.

- I have now expanded the search to include these two repositories

1. Please amend your detailed Financial Disclosure statement. This is published with the article, therefore should be completed in full sentences and contain the exact wording you wish to be published.

i). Please include all sources of funding (financial or material support) for your study. List the grants (with grant number) or organizations (with url) that supported your study, including funding received from your institution.

- The acknowledgement statement has been updated to read: “LA is supported by the National Institute for Health Research Applied Research Collaboration South West Peninsula (arc-swp.nihr.ac.uk). MA is an Academic Clinical Fellow supported by the National Institute for Health Research (nihr.ac.uk). The views expressed in this publication are those of the authors and not necessarily those of the National Institute for Health Research or the Department of Health and Social Care. The funders had no role in study design, data collection and analysis, decision to publish, or preparation of the manuscript.”

2. Please update your Competing Interests statement. If you have no competing interests to declare, please state: “The authors have declared that no competing interests exist.”

- I have updated the competing interests statement as advised

*Reviewer 1*

1. Dataset. What will be dataset available (if any) in this area. How to collect data by researchers themselves.

- The search details are available were registered with the Open Science Framework (DOI: 10.17605/OSF.IO/E7C2S). All the data extracted is detailed in the tables included within the manuscript. No is no other data to make available.

2. Experimental setup and performance metrics. What will be standard protocol to conduct experiments and what metrics should be used for quantitative performance evaluation of various methods.

- There was no standard protocol in the papers found. Details of metric used are detailed in the tables if quantitative measures were used. These were variable so no further analysis of these was possible.

3. Suggestion for future study. Any insightful suggestions for future research studies?

- I have added a section at the end of the discussion to highlight ideas for future research

4. Only two figures are provided in this 70-page paper, it is a bit dry. It would be good to include some figures to provide an overview of this research area.

- I have added extra figures as suggested

*Reviewer 2*

The review only included articles indexed in medical databases as opposed to purely technical databases such as The Institute of Electrical and Electronics Engineers (IEEE) Xplore Digital Library. Considering that the focus of the review is on technology, this omission may weaken the impact of this paper. Please consider justifying within the text the reason for this choice.

- Thank you for this feedback, as advised I have expanded the search to include IEEE Xplore and ACM Digital Library. The results of these searches have been included in the manuscript.
